# Supplementary material for: Impaired peroxisomal import in Drosophila oenocytes causes cardiac dysfunction by inducing upd3 as a peroxikine
Source: Nat Commun. 2020 Jun 10;11:2943. doi: 10.1038/s41467-020-16781-w (PMC7286907; doi:10.1038/s41467-020-16781-w)
Supplement: Supplementary file 1 — Supplementary information [file 41467_2020_16781_MOESM1_ESM.pdf]

Supplementary Information for

Impaired peroxisomal import in *Drosophila* hepatocyte-like cells causes cardiac dysfunction by inducing upd3 as a peroxikine

Kerui Huang[1], Ting Miao[1], Kai Chang[1], Jinoh Kim[1], Ping Kang[1], Qiuhan Jiang[1], Andrew J. Simmonds[2], Francesca Di Cara[3], Hua Bai[1]*

1 Department of Genetics, Development, and Cell Biology, Iowa State University, Ames, IA 50011, USA
2 Department of Cell Biology, University of Alberta, Edmonton, AB T6G 2H7, Canada.
3 Department of Microbiology & Immunology, Dalhousie University, Halifax, NS B3H4R2, Canada.

Supplementary Fig. 1

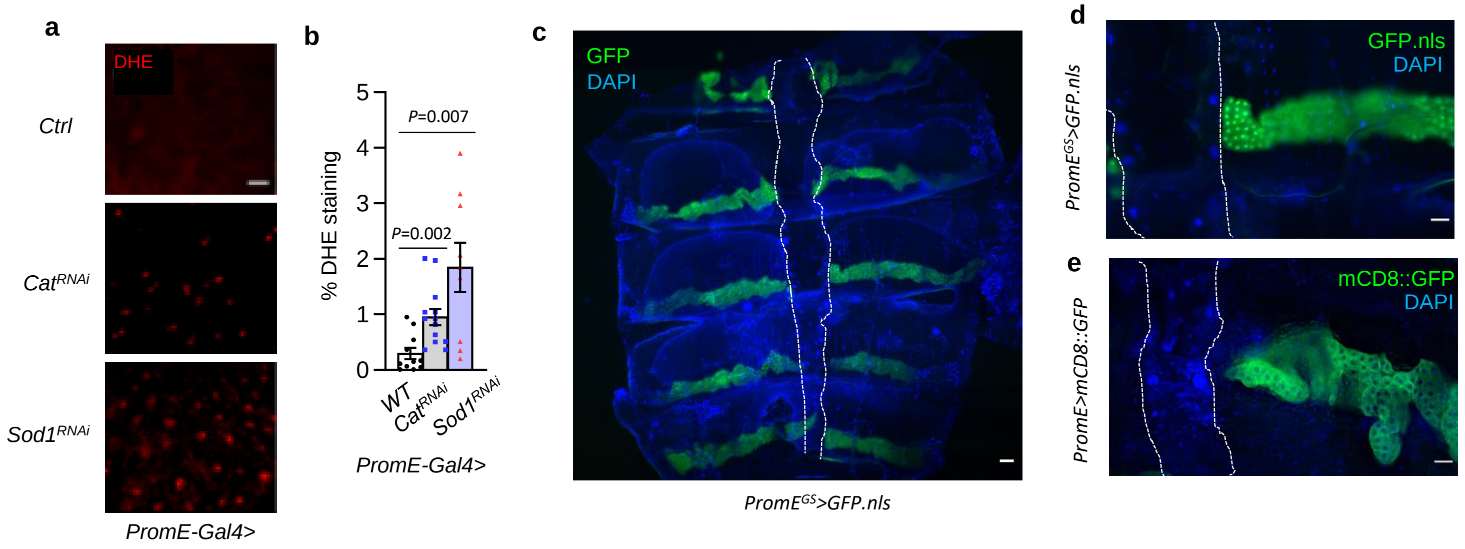

Supplementary Fig. 1 a DHE staining and PromE-gal4 specificity. Representative images to show DHE staining in oenocytes dissected from control and oenocyte-specific *Sod1* KD flies (*PromE-Gal4>UAS-Sod1*^{RNAi}). Hoechst 33342 was used for nuclear staining. Scale bar: 20 µm. **b** Quantification of relative DHE staining in control and oenocyte-specific *Sod1* KD flies (*PromE-Gal4>UAS-Cat*^{RNAi}). Data are represented as mean ± SEM. *P* values are calculated using one-way ANOVA followed by Holm-sidak multiple comparisons, ns: not significant. n = 5 flies, 2 ROI per replicate. **c, d, e** Verification of oenocyte-specific driver (*PromE-Gal4*), oenocyte-specific GeneSwitch driver (*PromE*^{GS}*-Gal4*). n = 5 flies. RU: mifepristone (RU486). Dashed line delineate cardiac cells. Scale bar: 20 µm. Source data are provided as a Source Data file.

Supplementary Fig. 2

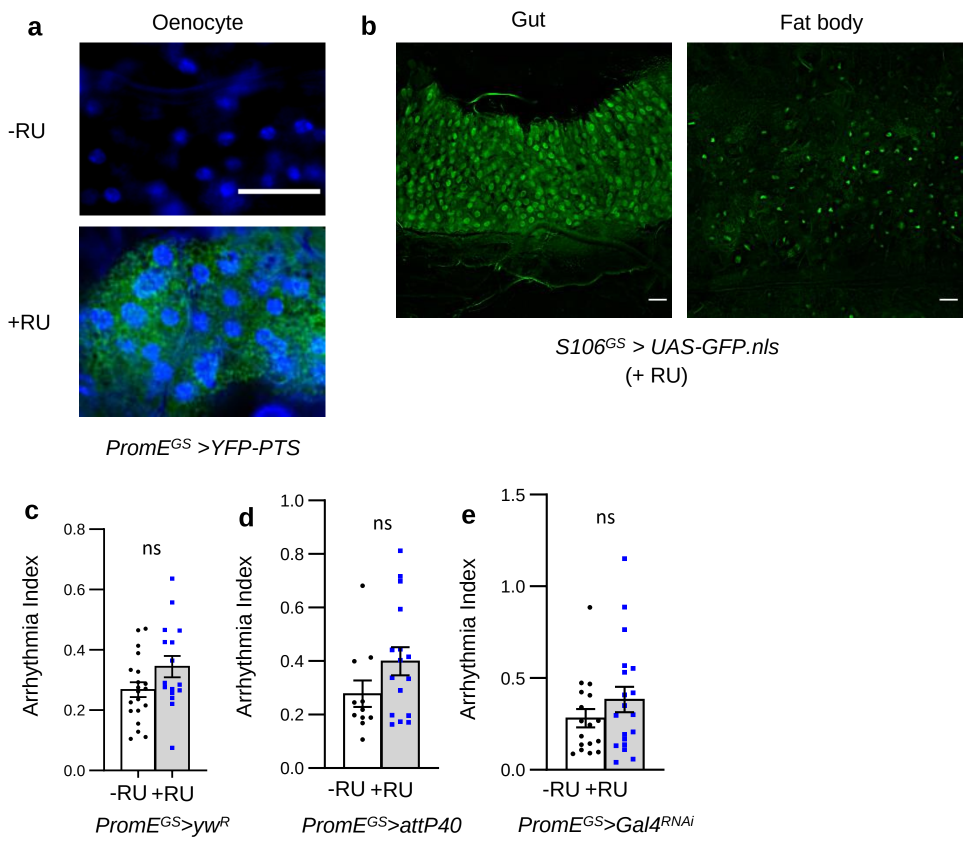

Supplementary Fig. 2 a Verification of oenocyte-specific GeneSwitch driver (*PromE^{GS}-Gal4*). RU: mifepristone (RU486). **b** Verification of fat body/gut-specific GeneSwitch driver (S106^{GS}-*Gal4*). Scale bar: 20 µm. n = 5 flies. Data presented here are representative of two independent experiments. **c, d, e** The effect of RU486 feeding on arrhythmia of three wild-type flies ($n_{left\text{-}right}$ = 20, 16, 11, 16, 17, 19 flies). *P* values are calculated using two-sided unpaired *t*-test. Source data are provided as a Source Data file. For specific statistical number, please refer to the source data.

Supplementary Fig. 3

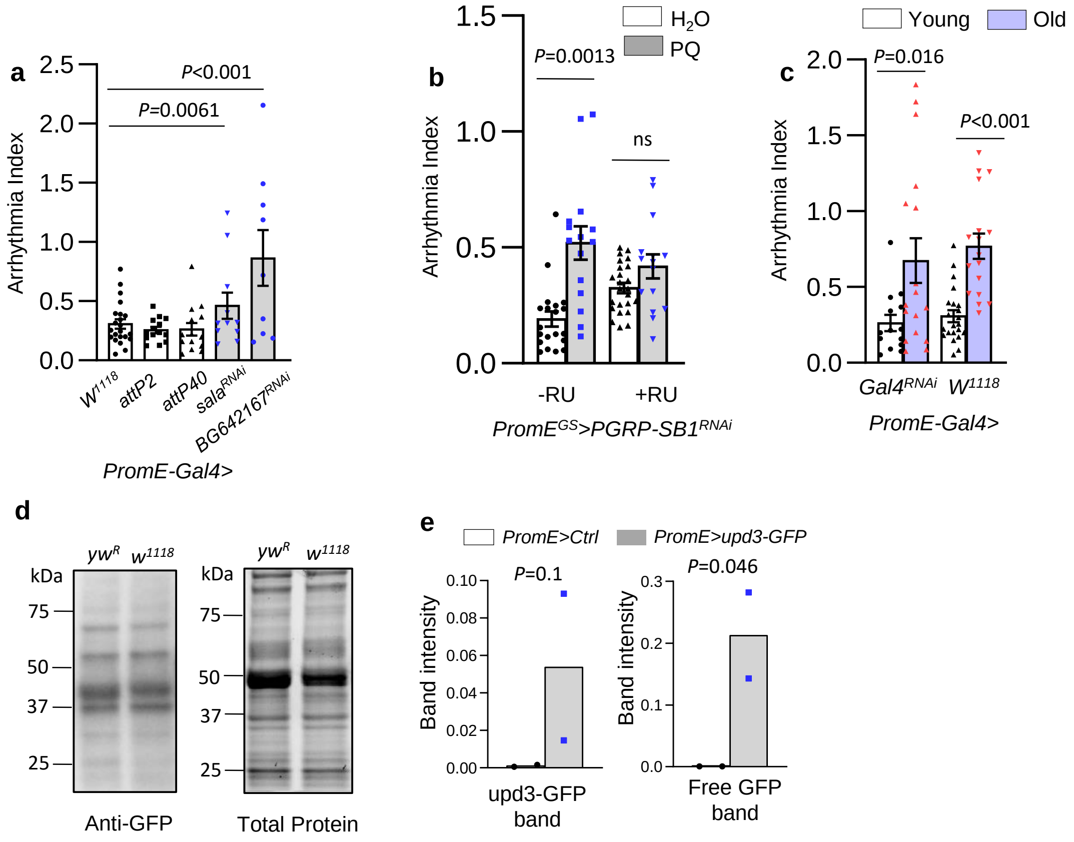

Supplementary Fig. 3a Arrhythmia index of wild-type flies (*PromE-Gal4>w^{1118}, PromE-Gal4>Attp2, PromE-Gal4>attP40*) and oenocyte-specific knockdown of *sala* and *BG642167* ($n_{left-right}$ = 14, 14, 22, 11, 9 flies). **b** Paraquat (PQ)-induced arrhythmia measured by SOHA for *PGRP-SB1* knockdown under oenocyte-specific GeneSwitch driver (*$PromE^{GS}$-Gal4>PGRP-$SB1^{RNAi}$*) ($n_{left-right}$ = 20, 15, 24, 15 flies). **c** Arrhythmia index of wild-type flies (*PromE-Gal4>UAS-$Gal4^{RNAi}$* and *PromE-Gal4>w^{1118}*) at young (2-week-old) and old age (6-week-old), ($n_{left-right}$ = 14, 17, 22, 17 flies). Data are represented as mean ± SEM. *P* values are calculated using one-way ANOVA, followed by Holm-sidak multiple comparisons ns: not significant. **d** Western blot analysis on the protein extracts from the whole body of two wild-type flies, *yw^R* and *w^{1118}*. Total protein loaded onto the Bio-Rad Stain-Free gel was visualized using ChemiDoc MP Imagers after UV activation. **e** Quantification of western blots in **Fig 3j**. The data represent the intensity of GFP bands normalized to the total protein. Source data are provided as a Source Data file. Data presented here are representative of two independent experiments. For specific statistical number, please refer to the source data.

Supplementary Fig. 4

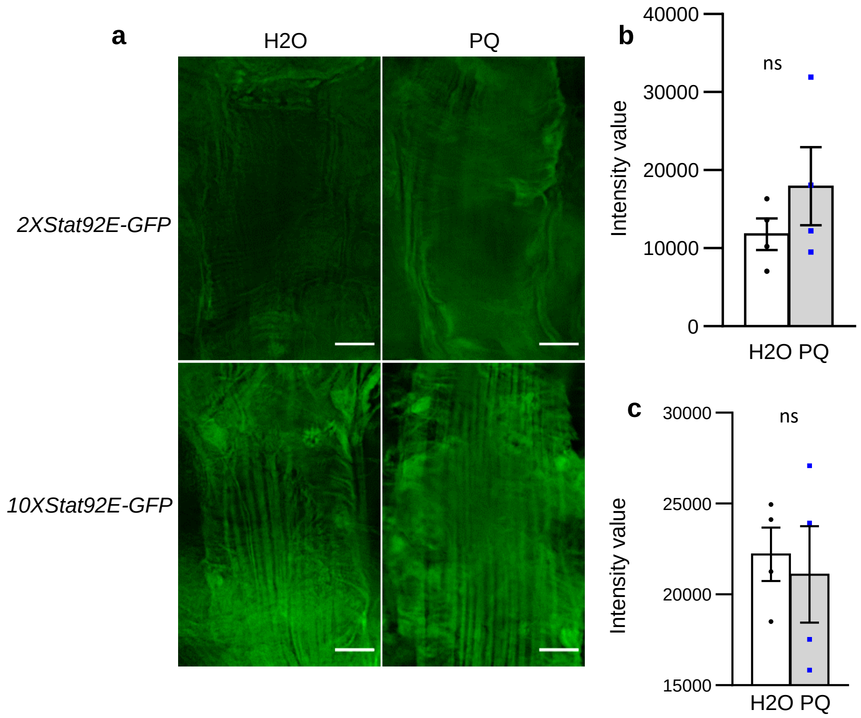

Supplementary Fig. 4 a Representative images of *2XStat92E-GFP* or *10XStat92E-GFP* reporters under paraquat feeding condition or water fed conditions. Scale bar: 20 µm. **b, c** Quantification of reporter staining in Panel, n = 4 flies. *P* values are calculated using two-sided unpaired *t*-test. **a.** Source data are provided as a Source Data file.

Supplementary Fig. 5

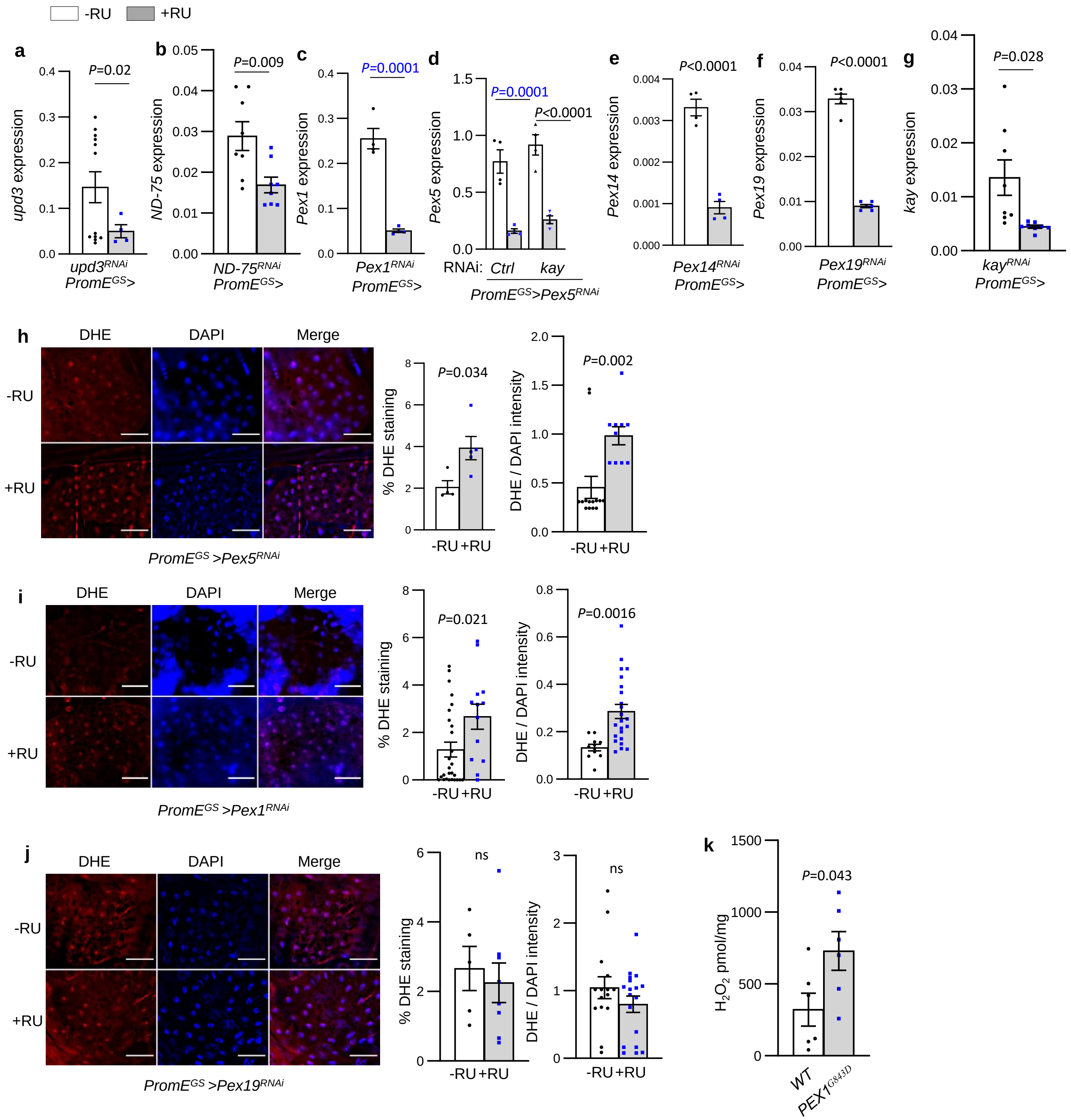

Supplementary Fig. 5 RNAi knockdown verification by QRT-PCR for *upd3* **(a)**, *ND-75* **(b)**, *Pex1* **(c)**, *Pex5* **(d)**, *Pex14* **(e)**, *Pex19* **(f)**, *kay* **(g)**. Oenocyte-specific GeneSwitch driver (*PromE*^{GS}*-Gal4*) was used. RU: mifepristone (RU486). **a-e, g** n = 4 biological samples. **f** n = 3 biological samples. ROS quantification in oenocyte-specific *Pex5* KD (**h**), *Pex1* KD (**i**), *Pex19* KD (**j**). % DHE-positive area per ROI or DHE intensity normalized to DAPI was analyzed.Scale bar: 20 µm. n = 4 flies. **k** H_2O_2 levels measured by Amplex Red in WT and PEX1-G843D human fibroblast cells. N = 6 biological samples. Data are represented as mean ± SEM. *P* values are calculated using unpaired *t*-test **(a-c, e-g, h-k)**, one-way ANOVA, followed by Holm-sidak multiple comparisons **(d).** ns: not significant. Source data are provided as a Source Data file. For statistical number, please refer to the source data.

Supplementary Fig. 6 Uncropped Original Scans

Fig3j:

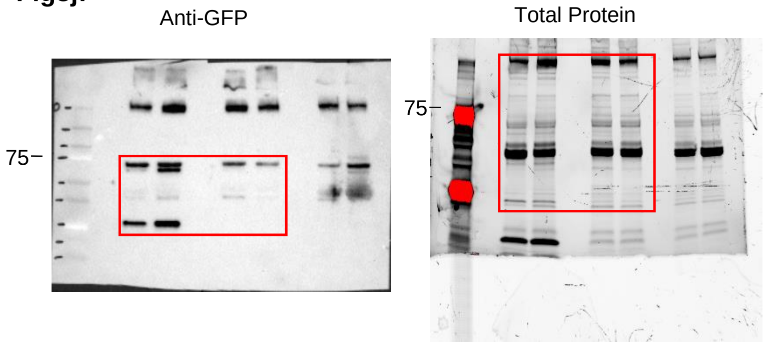

Fig5d:

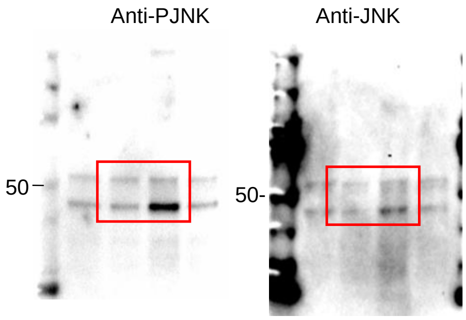

Fig5n:

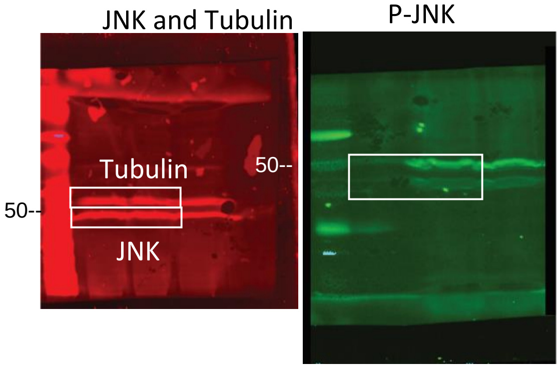

FigS3d:

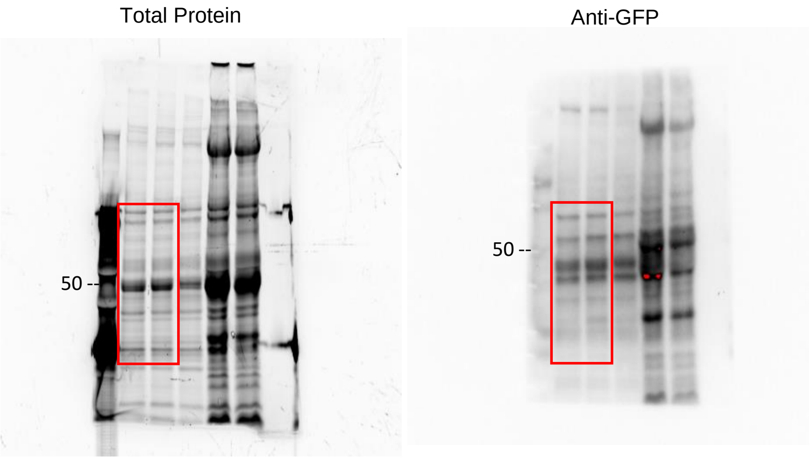

Supplementary Fig. 6 Uncropped and unprocessed gel or blots for **fig 3j, fig5d, 5n** and **supplementary fig3d.**

Supplementary Table 1: KEY RESOURCES TABLE

REAGENT OR RESOURCE	SOURCE	IDENTIFIER
**Antibodies**		
Rabbit anti-Stat92E	A gift from Steven X. Hou	N/A
Rabbit anti-GFP	Cell Signaling Technology	2956S
Guinea Pig anti-Pmp70	A gift from Kyu-Sun Lee	N/A
Rabbit anti-Pmp70	This study, by Andrew Simmonds	N/A
Rabbit anti-SKL	A gift from Richard Rachubinski	N/A
Rabbit anti-P-JNK	Cell Signaling Technology	4668S
Rabbit anti-P-JNK	Cell Signaling Technology	9255
Rabbit anti-JNK	Cell Signaling Technology	9252
Rabbit anti-Tubulin	Sigma	T5168
Goat anti-Rabbit IgG-HRP	Jackson ImmunoResearch	111-035-003
Alexa Fluor® 488 AffiniPure Donkey Anti-Rabbit IgG (H+L)	Jackson ImmunoResearch	711-545-152
Alexa Fluor® 594 AffiniPure Donkey Anti-Guinea Pig IgG (H+L)	Jackson ImmunoResearch	706-585-148
**Chemicals**		
Paraquat (PQ)	Sigma	36541-100M
Mifepristone (RU486)	Fisher Scientific	NC988828
16% Paraformaldehyde	Fisher Scientific	50980487
Hoechst 33342	ImmunoChemistry Technologies	N/A
ECL Western Blotting Substrate	Thermo Scientific	TD266065
2-Mercaptoethanol	Bio-Rad	161-0710
2X Laemmli sample buffer	Bio-Rad	161-0737
ProLong Gold antifade reagent	Fisher Scientific	S36937
**Critical Commercial Assays**		
Dihydroethidium (DHE)	Fisher Scientific	30980025MG
Cells-to-CT kits	Thermo Scientific	4402954
iScript™ cDNA Synthesis Kit	Bio-Rad	170-8891
Mini-PROTEAN TGX Stain-Free Precast Gels	Bio-Rad	456-8085
Amplex Red	Fisher Scientific	A22188
POWERUP SYBR GREEN MM	Fisher Scientific	A25778
**Experimental Models: Cell Lines**		
PEX1-G843D-PTS1	A gift from Nancy Braverman	N/A
Fibroblast cell line from healthy donors	A gift from Nancy Braverman	N/A
**Experimental Models (D.*melanogaster*): Strains**		
w*; PromE-gal4	Bloomington Drosophila Stock Center	65405
w*; PromE-gal4, mCD8::GFP	A gift from Alex Gould	N/A
yw; PromE-GS-gal4;+	A gift from Heinrich Jasper	N/A
w; +; PromE800-GS-gal4	A gift from Heinrich Jasper	N/A
w[1118]; P{w[+mC]=UAS-GFP.nls}14	Bloomington Drosophila Stock Center	4775
y1 v1; P{CaryP}attP40	Bloomington Drosophila Stock Center	36304
y1 v1; P{CaryP}attP2	Bloomington Drosophila Stock Center	36303
Gal4 RNAi: y[1] sc[*] v[1] sev[21]; P{y[+t7.7] v[+t1.8]=VALIUM20-GAL4.2}attP2	Bloomington Drosophila Stock Center	35783
w1118	A gift from Marc Tatar	N/A

ywR	A gift from Eric Rulifson	N/A
ywR;S106-GS-gal4;+	A gift from Marc Tatar	N/A
w; Hand4.2-gal4; +	A gift from Rolf Bodmer	N/A
w[1118]; P{w[+mC]=UAS-Sod1}12.1	Bloomington Drosophila Stock Center	33605
UAS-hop[tuml];+;+	A gift from Erika Bach	N/A
w; P{UAS-upd3-GFP}attp40	A gift from Doug Harrison	N/A
w[*]; P{w[+mC]=UAS-eYFP.PTS1}6	Bloomington Drosophila Stock Center	64248
hop RNAi: y[1] sc[*] v[1]; P{y[+t7.7] v[+t1.8]=TRiP.HMS00761}attP2	Bloomington Drosophila Stock Center	32966
Stat92E RNAi: y[1] v[1]; P{y[+t7.7] v[+t1.8]=TRiP.HMS00035}attP2	Bloomington Drosophila Stock Center	33637
dome RNAi: y[1] sc[*] v[1]; P{y[+t7.7] v[+t1.8]=TRiP.HMS01293}attP2	Bloomington Drosophila Stock Center	34618
dome RNAi: y[1] v[1]; P{y[+t7.7] v[+t1.8]=TRiP.HMJ21208}attP40	Bloomington Drosophila Stock Center	53890
Cat RNAi : y[1] sc[*] v[1]; P{y[+t7.7] v[+t1.8]=TRiP.HMS00990}attP2	Bloomington Drosophila Stock Center	34020
Sod1 RNAi : y[1] sc[*] v[1]; P{y[+t7.7] v[+t1.8]=TRiP.HMS01291}attP2	Bloomington Drosophila Stock Center	34616
CG11852 RNAi : y[1] sc[*] v[1] sev[21]; P{y[+t7.7] v[+t1.8]=TRiP.HMC04575}attP40	Bloomington Drosophila Stock Center	57193
CG13806 RNAi : y[1] sc[*] v[1] sev[21]; P{y[+t7.7] v[+t1.8]=TRiP.HMC04260}attP40	Bloomington Drosophila Stock Center	55965
CG34051 RNAi : y[1] sc[*] v[1] sev[21]; P{y[+t7.7] v[+t1.8]=TRiP.HMC05321}attP40	Bloomington Drosophila Stock Center	62848
NimB4 RNAi : y[1] sc[*] v[1] sev[21]; P{y[+t7.7] v[+t1.8]=TRiP.HMC04257}attP40	Bloomington Drosophila Stock Center	55963
lectin-46cb RNAi: y[1] v[1]; P{y[+t7.7] v[+t1.8]=TRiP.HMJ22160}attP40	Bloomington Drosophila Stock Center	58183
spz4 RNAi: y[1] sc[*] v[1] sev[21]; P{y[+t7.7] v[+t1.8]=TRiP.HMC05037}attP40	Bloomington Drosophila Stock Center	60044
sala RNAi: y[1] v[1]; P{y[+t7.7] v[+t1.8]=TRiP.HMJ22251}attP40	Bloomington Drosophila Stock Center	58231
upd2 RNAi: y[1] sc[*] v[1] sev[21]; P{y[+t7.7] v[+t1.8]=TRiP.HMS00948}attP2	Bloomington Drosophila Stock Center	33988
CG18628 RNAi: y[1] sc[*] v[1] sev[21]; P{y[+t7.7] v[+t1.8]=TRiP.HMC04437}attP40	Bloomington Drosophila Stock Center	56995
CG17633 RNAi: y[1] sc[*] v[1] sev[21]; P{y[+t7.7] v[+t1.8]=TRiP.HMC05036}attP40	Bloomington Drosophila Stock Center	60043
Npc2b RNAi: y[1] sc[*] v[1] sev[21]; P{y[+t7.7] v[+t1.8]=TRiP.HMS01682}attP40	Bloomington Drosophila Stock Center	38238
CG14259 RNAi: y[1] sc[*] v[1] sev[21]; P{y[+t7.7] v[+t1.8]=TRiP.HMC04578}attP40	Bloomington Drosophila Stock Center	57196
CG15201 RNAi: y[1] sc[*] v[1] sev[21]; P{y[+t7.7] v[+t1.8]=TRiP.HMC05343}attP40	Bloomington Drosophila Stock Center	62870
TotM RNAi: y[1] sc[*] v[1] sev[21]; P{y[+t7.7] v[+t1.8]=TRiP.HMC05034}attP40	Bloomington Drosophila Stock Center	60041
BthD RNAi: y[1] sc[*] v[1] sev[21]; P{y[+t7.7] v[+t1.8]=TRiP.HMC05991}attP40	Bloomington Drosophila Stock Center	65094
CecC RNAi: y[1] sc[*] v[1] sev[21]; P{y[+t7.7] v[+t1.8]=TRiP.HMC05727}attP40	Bloomington Drosophila Stock Center	64854

TotC RNAi: y[1] sc[*] v[1] sev[21]; P{y[+t7.7] v[+t1.8]=TRiP.HMC05166}attP2	Bloomington Drosophila Stock Center	62159
TotF RNAi: y[1] sc[*] v[1] sev[21]; P{y[+t7.7] v[+t1.8]=TRiP.HMC06169}attP40	Bloomington Drosophila Stock Center	65906
BG642167 RNAi: y[1] sc[*] v[1] sev[21]; P{y[+t7.7] v[+t1.8]=TRiP.HMC04592}attP40	Bloomington Drosophila Stock Center	57209
PGRP-SC2 RNAi: y[1] sc[*] v[1] sev[21]; P{y[+t7.7] v[+t1.8]=TRiP.HMC04353}attP40	Bloomington Drosophila Stock Center	56915
CG13618 RNAi: y[1] sc[*] v[1] sev[21]; P{y[+t7.7] v[+t1.8]=TRiP.HMC04942}attP40	Bloomington Drosophila Stock Center	57749
PGRP-SC1a RNAi: y[1] sc[*] v[1] sev[21]; P{y[+t7.7] v[+t1.8]=TRiP.HMC04566}attP40	Bloomington Drosophila Stock Center	57184
Notum RNAi: y[1] sc[*] v[1] sev[21]; P{y[+t7.7] v[+t1.8]=TRiP.HMC04067}attP40	Bloomington Drosophila Stock Center	55379
upd3 RNAi (#1): y[1] sc[*] v[1] sev[21]; P{y[+t7.7] v[+t1.8]=TRiP.HMS00646}attP2	Bloomington Drosophila Stock Center	32859
upd3 RNAi (#2): y[1] v[1]; P{y[+t7.7] v[+t1.8]=TRiP.HM05061}attP2	Bloomington Drosophila Stock Center	28575
TotA RNAi: y[1] sc[*] v[1] sev[21]; P{y[+t7.7] v[+t1.8]=TRiP.HMC04066}attP2	Bloomington Drosophila Stock Center	55378
Ag5r2 RNAi: y[1] sc[*] v[1] sev[21]; P{y[+t7.7] v[+t1.8]=TRiP.HMC04395}attP40	Bloomington Drosophila Stock Center	56955
PGRP-SB1 RNAi: y[1] sc[*] v[1] sev[21]; P{y[+t7.7] v[+t1.8]=TRiP.HMC04423}attP40	Bloomington Drosophila Stock Center	56983
Pex5 RNAi (#2): y[1] sc[*] v[1]; P{y[+t7.7] v[+t1.8]=TRiP.HMS02546}attP40	Bloomington Drosophila Stock Center	42854
Pex5 RNAi (#1): y[1] v[1]; P{y[+t7.7] v[+t1.8]=TRiP.HMJ21920}attP40	Bloomington Drosophila Stock Center	58064
Pex14 RNAi: y[1] sc[*] v[1]; P{y[+t7.7] v[+t1.8]=TRiP.HMC06491}attP40	Bloomington Drosophila Stock Center	77180
Pex1 RNAi: y[1] v[1]; P{y[+t7.7] v[+t1.8]=TRiP.HMC03252}attP2	Bloomington Drosophila Stock Center	51497
Pex19 RNAi: y[1] sc[*] v[1] sev[21]; P{y[+t7.7] v[+t1.8]=TRiP.HMC03104}attP2/TM3, Sb	Bloomington Drosophila Stock Center	50702
ND-75 RNAi (#1) : y[1] sc[*] v[1]; P{y[+t7.7] v[+t1.8]=TRiP.HMS00854}attP2	Bloomington Drosophila Stock Center	33911
ND-75 RNAi (#2): y[1] v[1]; P{y[+t7.7] v[+t1.8]=TRiP.JF02791}attP2	Bloomington Drosophila Stock Center	27739
Sod2 RNAi: y[1] v[1]; P{y[+t7.7] v[+t1.8]=TRiP.JF01989}attP2	Bloomington Drosophila Stock Center	25969
Prx5 RNAi: y[1] sc[*] v[1]; P{y[+t7.7] v[+t1.8]=TRiP.HMC05872}attP40	Bloomington Drosophila Stock Center	64998
Dhap-at RNAi (#1): y[1] sc[*] v[1]; P{y[+t7.7] v[+t1.8]=TRiP.HMC03654}attP40	Bloomington Drosophila Stock Center	52914
Dhap-at KO (#2): y[1] sc[*] v[1] sev[21]; P{y[+t7.7] v[+t1.8]=TKO.GS00835}attP40	Bloomington Drosophila Stock Center	77048
ADPS RNAi: y1 sc* v1 sev21; P{TRiP.HMS01339}attP2	Bloomington Drosophila Stock Center	34350
Acox57D-d RNAi: y[1] v[1]; P{y[+t7.7] v[+t1.8]=TRiP.HMJ30208}attP40	Bloomington Drosophila Stock Center	63641
Acox57D-p RNAi: y[1] sc[*] v[1]; P{y[+t7.7] v[+t1.8]=TRiP.HMC05604}attP40	Bloomington Drosophila Stock Center	64585

kay RNAi: y[1] sc[*] v[1]; P{y[+t7.7] v[+t1.8]=TRiP.HMS00254}attP2	Bloomington Drosophila Stock Center	33379
Jra RNAi: y[1] v[1]; P{y[+t7.7] v[+t1.8]=TRiP.JF01184}attP2	Bloomington Drosophila Stock Center	31595
UAS-dCAS9-VPR: w[*]; P{w[+mC]=UAS-3xFLAG.dCas9.VPR}attP40; P{GAL4-Mhc.W}MHC-82/TM6B, Tb[1]	Bloomington Drosophila Stock Center	67044
Pex5 gRNA: y[1] sc[*] v[1] sev[21]; P{y[+t7.7] v[+t1.8]=TOE.GS01923}attP40	Bloomington Drosophila Stock Center	78666
2XStat92E-GFP: w[1118]; P{w[+mC]=2XStat92E-GFP}6-1	Bloomington Drosophila Stock Center	26196
10XSTAT92E-GFP on (II) and (III) chromosome	Douglas A Harrison	N/A
TRE-DsRedT4 reporter: w[*]; P{y[+t7.7] w[+mC]=TRE-DsRedT4}attP40	Bloomington Drosophila Stock Center	59011
**Oligonucleotides (see Table S2 for sequences)**		
RpL32	This paper	N/A
upd1	This paper	N/A
upd2	This paper	N/A
upd3	This paper	N/A
puc	This paper	N/A
yki	This paper	N/A
Mad	This paper	N/A
kay	This paper	N/A
Jra	This paper	N/A
Socs36E	This paper	N/A
Pex5	This paper	N/A
Pex1	This paper	N/A
Pex14	This paper	N/A
Pex19	This paper	N/A
ND-75	This paper	N/A
IL-6	This paper	N/A
GAPDH	This paper	N/A
**Others**		
Borosilicate Glass Capillary Tubes	WPI	#1B100F-4
Sutter Puller	Sutter	Model P-97
Hamamatsu ORCA-Flash 4.0 digital camera	Hamamatsu	ORCA-Flash 4.0
Olympus BX51WI upright microscope	Olympus	BX51WI
Olympus FV3000 Confocal Laser Scanning Microscope	Olympus	FV3000
Quantstudio 3 Real-Time PCR System	Thermo Fisher Scientific	Quantstudio 3 (version 1.2.1)
**Software**		
ImageJ	NIH Image	Version 1.49
HCI imaging software	Hamamatsu Photonics	Version 4.6.1
GraphPad Prism	Prism	Version 6.07
Bio-Rad ChemiDoc	Bio-Rad	Version 6.0.1
CellSens	Olympus	Version 1.16
SOHA	Developed by Rolf Bodmer and Karen Ocorr	x86

Supplementary Table 2: Primer list

**Oligonucleotides**	**Sequence 5'-3'**	**Species**
upd1 F	CGCAGCCTAAACAGTAGCCA	*Drosophila melanogaster*
upd1 R	CGCTTTAGGGCAATCGTGGA	*Drosophila melanogaster*
upd2 F	CTTAAACGCCAGCCAACAGAG	*Drosophila melanogaster*
upd2 R	TGAATGGCATCACGACGCT	*Drosophila melanogaster*
upd3[#1] F	AAAACGGCCAGAACCAGGAA	*Drosophila melanogaster*
upd3[#1] R	CATGGCCAAGGCGAGTAAGA	*Drosophila melanogaster*
upd3[#2] F	AATGCCAGCAGTACGCATCT	*Drosophila melanogaster*
upd3[#2] R	TTCTGCAGGATCCTTTGGCG	*Drosophila melanogaster*
puc F	ATCGAAGATGCACGGAAAAC	*Drosophila melanogaster*
puc R	CAGGGAGAGCGACTTGTACC	*Drosophila melanogaster*
yki F	AACTAGGCGCCTTGCCG	*Drosophila melanogaster*
yki R	TCGCTCGGCCATCAAGATTT	*Drosophila melanogaster*
Mad F	GTGCGTGTGAGTGAAAGCTA	*Drosophila melanogaster*
Mad R	GGTATTGGAGTAGCTGCCGT	*Drosophila melanogaster*
kay F	CGCAACATTGCGCTATTTTCAA	*Drosophila melanogaster*
kay R	GCTTTTGTTGAATCGTTTTGGGT	*Drosophila melanogaster*
Jra F	ATTCCGCCGCCAATAACA	*Drosophila melanogaster*
Jra R	CTCGTCCTTAATCACCGAGAAG	*Drosophila melanogaster*
Socs36E F	AGTCGCAGCAGTAAAGCACT	*Drosophila melanogaster*
Socs36E R	TTAATCCTCGGATGGCGTCG	*Drosophila melanogaster*
Pex5 F	CAACCTTACACACCCACATGAC	*Drosophila melanogaster*
Pex5 R	GCAGCGATCTCCAGAGTTAT	*Drosophila melanogaster*
Pex1 F	GATCTGGTCAAGTGTGCGCT	*Drosophila melanogaster*
Pex1 R	AGCACACTGCCCGATATCTTT	*Drosophila melanogaster*
Pex14 F	CATTGGATCCCAATTGCACGC	*Drosophila melanogaster*
Pex14 R	AACAGGTAGGGCGCAATGTA	*Drosophila melanogaster*
Pex19 F	TTCATGGAGGGCATGATGCAG	*Drosophila melanogaster*
Pex19 R	TGTCCTCAGCGGAGAGCTT	*Drosophila melanogaster*
ND-75 F	GTACCCGGCACCACTGTC	*Drosophila melanogaster*
ND-75 R	AGCAGAATCTGGGTATCTCCAC	*Drosophila melanogaster*
RpL32 F	AAGAAGCGCACCAAGCACTTCATC	*Drosophila melanogaster*
RpL32 R	TCTGTTGTCGATACCCTTGGGCTT	*Drosophila melanogaster*
IL-6 F	CCACACAGACAGCCACTCA	*Homo sapiens*
IL-6 R	CATCCATCTTTTTCAGCCATCT	*Homo sapiens*
GAPDH F	ACCCACTCCTCCACCTTTG	*Homo sapiens*
GAPDH R	CTCTTGTGCTCTTGCTGGG	*Homo sapiens*
