# Supplementary material for: Impaired peroxisomal import in Drosophila oenocytes causes cardiac dysfunction by inducing upd3 as a peroxikine
Source: Nat Commun. 2020 Jun 10;11:2943. doi: 10.1038/s41467-020-16781-w (PMC7286907; doi:10.1038/s41467-020-16781-w)
Supplement: Supplementary file 3 — Reporting Summary [file 41467_2020_16781_MOESM3_ESM.pdf]

nature research

Corresponding author(s): Hua Bai

Last updated by author(s): Apr 23, 2020

Reporting Summary

Nature Research wishes to improve the reproducibility of the work that we publish. This form provides structure for consistency and transparency in reporting. For further information on Nature Research policies, see Authors & Referees and the Editorial Policy Checklist .

Statistics

For all statistical analyses, confirm that the following items are present in the figure legend, table legend, main text, or Methods section.

n/a	Confirmed	
☐	☒	The exact sample size (*n*) for each experimental group/condition, given as a discrete number and unit of measurement
☐	☒	A statement on whether measurements were taken from distinct samples or whether the same sample was measured repeatedly
☐	☒	The statistical test(s) used AND whether they are one- or two-sided<br>*Only common tests should be described solely by name; describe more complex techniques in the Methods section.*
☒	☐	A description of all covariates tested
☐	☒	A description of any assumptions or corrections, such as tests of normality and adjustment for multiple comparisons
☐	☒	A full description of the statistical parameters including central tendency (e.g. means) or other basic estimates (e.g. regression coefficient) AND variation (e.g. standard deviation) or associated estimates of uncertainty (e.g. confidence intervals)
☐	☒	For null hypothesis testing, the test statistic (e.g. *F*, *t*, *r*) with confidence intervals, effect sizes, degrees of freedom and *P* value noted<br>*Give P values as exact values whenever suitable.*
☒	☐	For Bayesian analysis, information on the choice of priors and Markov chain Monte Carlo settings
☒	☐	For hierarchical and complex designs, identification of the appropriate level for tests and full reporting of outcomes
☐	☒	Estimates of effect sizes (e.g. Cohen's *d*, Pearson's *r*), indicating how they were calculated

Our web collection on statistics for biologists contains articles on many of the points above.

Software and code

Policy information about availability of computer code

Data collection	CellSense Dimensions software (Olympus, version 1.16) was used for image acquisition; Quantstudio 3 Real-Time PCR system was used for RT-PCR<br>analysis (version 1.2.1); Semi-Automated Optical Heartbeat Analysis (SOHA) was used to measure cardiac function (x86); Bio-Rad ChemiDoc (version 6.0.1) was used to<br>obtain western blot images.
Data analysis	CellSense Dimensions software (Olympus, version 1.16) was used for quantification and deconvolution; ImageJ was used for quantification and colocalization<br>analysis (v 1.49); GraphPad Prism 6.07 was used for data visualization. HCI imaging software (v 4.6.1).

For manuscripts utilizing custom algorithms or software that are central to the research but not yet described in published literature, software must be made available to editors/reviewers. We strongly encourage code deposition in a community repository (e.g. GitHub). See the Nature Research guidelines for submitting code & software for further information.

Data

Policy information about availability of data

All manuscripts must include a data availability statement. This statement should provide the following information, where applicable:

- Accession codes, unique identifiers, or web links for publicly available datasets
- A list of figures that have associated raw data
- A description of any restrictions on data availability

Full scans of the gels and blots are available in Supplementary Figs. 6. Source data underlying (Figs. 1, figs. 2b-k, Figs 3-7 and Supplementary Figs. 1-5 are provided as a Source Data file. For RNA-seq results on aging and paraquat treated oenocytes (Fig 2a), see https://bmcgenomics.biomedcentral.com/articles/10.1186/s12864-018-5404-4, Additional File 2.

Field-specific reporting

Please select the one below that is the best fit for your research. If you are not sure, read the appropriate sections before making your selection.

☒ Life sciences ☐ Behavioural & social sciences ☐ Ecological, evolutionary & environmental sciences

For a reference copy of the document with all sections, see nature.com/documents/nr-reporting-summary-flat.pdf

Life sciences study design

All studies must disclose on these points even when the disclosure is negative.

Sample size	Sample size was stated as the number of fruit flies used in each experiment. For fly cardiac function, samples size is 9-50, determined according to previously published relevant papers (Clara Guida, 2019; Chang, 2019; Nishimura, 2014; Fink, 2009). Immunostaining and staining on fly tissues, sample size is 6, as determined from previous immunostaining studies using Drosophila (Zhao, 2019; Huang, 2019; Chang, 2019). For Real Time PCR analysis, sample size was determined as the number of biological replicates (sample size = 3-4) according to previously published study (Owusu-Ansah, 2013; Di Cara, 2017). Each biological replicate contains cDNA from 10 flies to maintain a stable Ct value Rpl32 around 21.
Data exclusions	Outliers are excluded and identified using ROUT method (Motulsky, 2006) from GraphPad, with Q = 1%. Outliers were removed to eliminate the possibility of human error during dissection. The method was previously established in similar studies (Clara Guida, 2019; Chang, 2019; Wang, 2011; Steinert, 2014).
Replication	Most of the cardiac function experiments were confirmed by using different lines of RNAi and at least two independent experiments were performed. For Real Time PCR analysis at least two independent experiments were performed. Most of the immunostaining results were from two independent experiments. All attempts to replicate were successful.
Randomization	Fruit fly progenies from certain genotype are collected. Only female progeny is selected for following experiments. Female flies with correct genotype are randomly selected to perform experiments.
Blinding	For image analysis, investigators were blinded to group allocation during data collection and analysis. For cardiac function analysis and PCR, only single blind method was followed because the experiments allow little personal interpretation, as data is quantified by Software automatically.

Reporting for specific materials, systems and methods

We require information from authors about some types of materials, experimental systems and methods used in many studies. Here, indicate whether each material, system or method listed is relevant to your study. If you are not sure if a list item applies to your research, read the appropriate section before selecting a response.

Materials & experimental systems

n/a	Involved in the study	
☐	☒	Antibodies
☐	☒	Eukaryotic cell lines
☒	☐	Palaeontology
☐	☒	Animals and other organisms
☒	☐	Human research participants
☒	☐	Clinical data

Methods

n/a	Involved in the study	
☒	☐	ChIP-seq
☒	☐	Flow cytometry
☒	☐	MRI-based neuroimaging

Antibodies

Antibodies used

1. Anti-STAT92E: generated from Steven X. Hou;
2. anti-GFP, Cell Signaling, ID: 2956S;
3. anti-PMP70 (Guinea Pig), generated from Kyu-Sun Lee;
4. anti-PMP70 (Rabbit), generated from Andrew Simmonds U of A;
5. anti-SKL (Rabbit), generated from Richard Rachubinski U of A;
6. anti-P-JNK, Cell Signaling, ID: 4668S;
7. anti-P-JNK, Cell Signaling, ID: 9255;
8. anti-JNK, Cell Signaling, ID: 9252;
9. anti-Tubulin, Sigma, ID: T5168.
10. Goat anti-Rabbit IgG-HRP, Jackson ImmunoResearch, 111-035-003
11. Alexa Fluor® 488 AffiniPure Donkey Anti-Rabbit IgG (H+L), Jackson ImmunoResearch, 711-545-152

	12. Alexa Fluor® 594 AffiniPure Donkey Anti-Guinea Pig IgG (H+L), Jackson ImmunoResearch, 706-585-148
Validation	1. Anti-STAT92E validated in previous literature (Hua-Wei Chen, 2002);<br>2. Antibody verified using western blot analysis from HCC82 cells on company website and verified in flies using western blot;<br>3. Verified from unpublished study from Kyu-Sun Lee;<br>4. Generated against Drosophila PMP70 region 646-665, from Pacific Immunology;<br>5. Verified in published literature (Di cara, 2019 ; Szilard, 1995);<br>6. Verified using western blot from 293 cells and C6 cells, recognition sequence is highly conserved with Drosophila, and it has species reactivity in Drosophila according to antibody profile in online databases;<br>7. Verified using western blot from 293, it has species reactivity in Drosophila according to antibody profile in online databases (de Vreede, 2018);<br>8. Verified using western blot from 293 and SK-N-MC cells, it has species reactivity in Drosophila according to antibody profile in online databases (Koon, 2018);<br>9. Verified using western blot and it has reactivity in Drosophila according to online databases (Weiss, 2019; Kim, 2019).

Eukaryotic cell lines

Policy information about cell lines

Cell line source(s)	The human PEX1-G843D-PTS1 and wild type cell lines were gifts from the Richard Rachubinski (Zhang et al., 2010). The cell line was established from patients with PEX1-p.G843D. The wild-type cell lines were established from the fibroblasts of healthy donors.
Authentication	Both cell lines were originally developed by Richard Rachubinski in a collaboration (Zhang et al., 2010). No further authentication was performed.
Mycoplasma contamination	PCR amplification detected no mycoplasma genomes.
Commonly misidentified lines (See ICLAC register)	No commonly misidentified lines were used in this study.

Animals and other organisms

Policy information about studies involving animals; ARRIVE guidelines recommended for reporting animal research

Laboratory animals	Drosophila Melanogaster, 14-day-old to 42-day-old females.
Wild animals	This study did not involve wild animals.
Field-collected samples	This study did not involve field-collected samples.
Ethics oversight	No ethical approval was required.

Note that full information on the approval of the study protocol must also be provided in the manuscript.
